# Combination of generalist predators, *Nesidiocoris tenuis* and *Macrolophus pygmaeus*, with a companion plant, *Sesamum indicum*: What benefit for biological control of *Tuta absoluta*?

**DOI:** 10.1371/journal.pone.0257925

**Published:** 2021-09-30

**Authors:** Kouassi Arthur J. Konan, Lucie S. Monticelli, San-Whouly M. Ouali-N’goran, Ricardo Ramirez-Romero, Thibaud Martin, Nicolas Desneux

**Affiliations:** 1 Felix Houphouet Boigny University of Cocody, Abidjan, Côte d’Ivoire; 2 National Center for Agricultural Research (CNRA), Bouaké, Côte d’Ivoire; 3 INRAE, CNRS, UMR ISA, Université Côte d’Azur, Nice, France; 4 CUCBA, Universidad de Guadalajara, Zapopan, Jalisco, Mexico; 5 Cirad UR Hortsys, Abidjan, Côte d’Ivoire; University of Carthage, TUNISIA

## Abstract

*Tuta absoluta* is one of the most damaging pests of tomato crops worldwide. Damage due to larvae may cause up to 100% loss of tomato production. Use of natural enemies to control the pest, notably predatory mirids such as *Nesidiocoris tenuis* and *Macrolophus pygmaeus*, is increasingly being promoted. However, considering the potential damage caused to tomatoes by these omnivorous predators in the absence of *T*. *absoluta*, an alternative solution could be required to reduce tomato damage and improve the predators’ performance. The use of companion plants can be an innovative solution to cope with these issues. The present study aimed to determine the influence of companion plants and alternative preys on the predators’ performance in controlling *T*. *absoluta* and protecting tomato plants. We evaluated the effect of predators (alone or combined) and a companion plant (sesame (*Sesamum indicum*)) on *T*. *absoluta* egg predation and crop damage caused by *N*. *tenuis*. The influence of an alternative prey (*Ephestia kuehniella* eggs) on the spatial distribution of predators was also evaluated by caging them in the prey presence or absence, either on tomato or sesame plants or on both. We found that the presence of sesame did not reduce the efficacy of *N*. *tenuis* or *M*. *pygmaeus* in consuming *T*. *absoluta* eggs; hatched egg proportion decreased when *N*. *tenuis*, *M*. *pygmaeus*, or both predators were present. More specifically, this proportion was more strongly reduced when both predators were combined. Sesame presence also reduced necrotic rings caused by *N*. *tenuis* on tomato plants. *Nesidiocoris tenuis* preferred sesame over tomato plants (except when food was provided only on the tomato plant) and the upper part of the plants, whereas *M*. *pygmaeus* preferred tomato to sesame plants (except when food was provided only on the sesame plant) and had no preference for a plant part. Combination of predators *N*. *tenuis* and *M*. *pygmaeus* allows for better coverage of cultivated plants in terms of occupation of different plant parts and better regulation of *T*. *absoluta* populations. *Sesamum indicum* is a potential companion plant that can be used to significantly reduce *N*. *tenuis* damage to tomatoes.

## Introduction

The South American tomato pinworm, *Tuta absoluta* Meyrick (Lepidoptera: Gelechiidae), is a key pest of tomato crops (*Solanum lycopersicum* L.) worldwide [1–7]. In the absence of effective management methods, serious damage owing to larval feeding activity [8–10] can lead to up 80–100% production loss [1]. In general, the spraying of chemical insecticides in high quantities remains a preferred pest management method and ensures better tomato production [11, 12]. Unfortunately, this practice promotes selection of resistant pest populations [13, 14], can generate detrimental side effects on the environment, affects beneficial arthropods [15–20] and is not always effective against *T*. *absoluta* because of resistance developed to several active substances [21, 22]. The development of more appropriate methods for better management of *T*. *absoluta* is increasingly becoming a priority [1, 2].

Integrated management programs against *T*. *absoluta* seek to keep damage below the level of economic damage to tomatoes. A key component of such programs is biological control [1, 23]. Among the possibilities of biological control, a number of natural enemies proved to reduce pest damage in glasshouse tomato crops [24–26]. Biological control practices have long emphasized the role of specialized natural enemies, whose dynamics are closely related to those of a target pest [27, 28]. However, the presence of pest complexes in the agroecosystems limits the effectiveness of specialized predators. For this reason, there has been a growing interest for generalist predators because of their high adaptation and widespread success compared to other more specialized natural enemies [29].

Generalist predators, e.g. *Macrolophus pygmaeus* Rambur, *Nesidiocoris tenuis* Reuter (Hemiptera: Miridae), *Dicyphus tamaninii* Wagner, and *D*. *errans* Wolff, are considered important biological control agents against several tomato crop pests such as mites, whiteflies, thrips, aphids, and pinworms [2, 8, 30, 31]. These species often coexist in agroecosystems (e.g., *M*. *pygmaeus* and *N*. *tenuis*) [32] and are able to switch successfully to the predation of the eggs of *T*. *absoluta*, an invasive pest, shortly after its introduction in the Mediterranean region [31–35]. However, *M*. *pygmaeus* is not always effective when used alone against *T*. *absoluta* because it seems to have other preferences (for example whiteflies) [33, 34]. As for *N*. *tenuis*, it causes necrotic rings due to its repeated feeding around stems and flowers often requiring the use of insecticide to reduce population density [36, 37].

In such a context, the combination of companion or service plants with predators has become a newly adopted option to optimize the efficacy of the latter and reduce *N*. *tenuis* damage on cultivated plants. Companion or service plants can serve as an appropriate refuge when the crop environment is unfavorable, for example, in the absence of prey or pesticide use [38, 39]. They could act as a mini-breeding system in fields [38], diversify habitats, reduce the frequency of encounters between predators and thus reduce antagonistic effects [40]. Therefore, special attention should be paid to companion plants such as the sesame plant *Sesamum indicum* L. (Pedaliaceae) in pest management systems. With or without prey, *N*. *tenuis* prefers this plant for its reproduction rather than tomato, cucumber, eggplant, or pepper [41]. In the presence of sesame, *N*. *tenuis* causes much less damage to tomato plants while preying on *T*. *absoluta* eggs [42]. The combined action of natural enemies can have synergistic or additive effects that enhance pest control [43–47]. Antagonistic effects can also be observed when natural enemies share the same prey [48]. Sesame as a companion plant is a potential option to be evaluated with *N*. *tenuis* and *M*. *pygmaeus* both in the laboratory and in agricultural systems in order to limit the phytophagy of *N*. *tenuis* on tomato crops. If confirmed, such a system’s effectiveness could enhance integrated pest management programs. Some authors [49] still believe that an effective biocontrol agent must be species-specific to the prey or host. However, an assemblage of generalist predators can be effective in decreasing populations densities of both native and exotic pest species [50–52].

In this assemblage, the impact of the alternative prey should be considered, as this factor can negatively or positively influence predator behavior and pest control [34, 53, 54]. Alternative prey may divert predation pressure by reducing the risk of predation on the focal prey [55, 56] depending on predator preferences [53, 54]. Conversely, predation on the alternative prey may stimulate predator populations to respond numerically and consume more individuals of the target prey [54, 57]. Thus, the presence of alternative prey allows generalist predators to rapidly establish themselves in agroecosystems before the arrival of pests [54, 58]. This limits the growth of pest populations after they have colonized the crop [59].

Evaluation of such a system involves monitoring the behavior of associated individuals (i.e., *N*. *tenuis* and *M*. *pygmaeus*), including monitoring their distribution within plants, their preference, intraguild predation (IGP), etc.

In this study, the objectives were to assess the effect of a companion plant (sesame) and the use of two predators (i) on the predation rates of *T*. *absoluta* eggs, (ii) on *N*. *tenuis* damage to tomato plants and (iii) to determine the influence of an alternative prey (lepidopteran eggs) on the spatial distribution of the predators.

We expected that (1) the combination of both predators in the presence of sesame would increase *T*. *absoluta* egg predation, (2) the presence of companion plants would diversify habitats and reduce the phytophagy of *N*. *tenuis* on tomato plants and (3) the presence of alternative prey would not affect the behavior of predators in relation to their distribution within plants.

## Materials and methods

### Biological material

Tomato (cv. Nano) and sesame (cv. T-85 Humera) plants used for insect rearing and experiments were planted in plastic pots (9 x 9 x 10 cm) in the laboratory. They were maintained in controlled conditions (24 ± 2°C, 40 ± 10% R.H. and 16:8 L:D) until they reached a height of 15 to 20 cm (4- to 5-weeks-old).

A *T*. *absoluta* colony was maintained on young tomato plants in laboratory (24 ± 2°C, 40 ± 10% R.H. and 16:8 L:D). It originated from 65 individuals collected in 2009 on greenhouse tomato plants in the South of France, and at least 50 individuals collected in tomato fields were added yearly. *Tuta absoluta* eggs were obtained by introducing eight tomato plants in cages and adding five adult pairs of the pinworm for 48 hours in the cages. The eggs laid were then counted, and 40 were kept per plant. Plants with eggs were moved to new cages holding predators and/or companion plants.

The predators *N*. *tenuis* and *M*. *pygmaeus* were provided by Koppert Biological Systems, France and reared on a tomato plant in cages covered with a fine nylon mesh (30 x 30 x 60 cm). Predators were fed with eggs of *E*. *kuehniella* Zeller (Lepidoptera: Pyralidae) and diluted honey. The *E*. *kuehniella* eggs were replaced every two days and provided as libitum. All the experiments were carried out at the French National Institute of Agronomic, Food and Environment Research (INRAE), Sophia Antipolis in the same laboratory conditions (24 ± 2°C, 40 ± 10% R.H. and 16:8 L:D).

### Influence of a companion plant and predators on *Tuta absoluta* predation and tomato protection

The predator activity was determined by the ability of *N*. *tenuis* and *M*. *pygmaeus* to reduce the *T*. *absoluta* egg hatching rate after 9 days in the presence or absence of a sesame plant (S). In this experiment, tomato plants with *T*. *absoluta* eggs were used in all treatments. The proportion of hatched eggs on tomato plants was therefore assessed according to the presence of both predators combined (N-M) or individually (N or M) and with or without the companion plant (S). Thus 7 combinations were made with tomato plants containing *T*. *absoluta* eggs in different cages (30 x 30 x 60 cm covered with fine nylon mesh). These treatments were composed of (1) both predators with sesame (N-M&S); (2) both predators without sesame (N-M); (3) *N*. *tenuis* with sesame (N&S); (4) *M*. *pygmaeus* with sesame (M&S); (5) *N*. *tenuis* without sesame (N); (6) *M*. *pygmaeus* without sesame (M) and (7) sesame (S).

Each treatment involved 6 repetitions with one control at each repetition (the sesame plant only). A total of 48 tomato plants and 32 sesame plants were used. For treatments with both predator species, one couple of each species was released in the cages. In contrast, treatments with only one species included two couples of that species. Forty-eight hours later, predators were removed from the different cages. Egg hatching was monitored during 9 days and eggs that did not hatch during this period were considered consumed by the predators [42]. The number of *T*. *absoluta* larvae was counted to determine the hatching reduction. The hatching reduction rate was calculated by comparing the number of larvae that emerged with the initial number of eggs (40 eggs), corrected by the average egg mortality observed in the control according to the formula: RE = 100 × [1-(Ex/Et)] where Ex is the average number of eggs hatched during the treatment and Et is the average number of eggs hatched in the control.

### *Nesidiocoris tenuis* phytophagy

The phytophagy on the tomato plants was assessed by counting the number of necrotic rings on the main stem, young shoots, leaves and leaf petioles [37, 42] induced by *N*. *tenuis*. The number of necrotic rings was assessed in the presence or absence of the second predator *M*. *pygmaeus* (M) and with or without the sesame plant (S) as a companion plant. The necrotic rings counts were made 3 days after the predators were removed from the cages. Six replicates with a control at each replicate (sesame plant only) were performed for each treatment. A total of 48 tomato plants and 32 sesame plants were used to assess the phytophagy. The experiments were carried out at 24 ± 2°C, 40 ± 10% R.H. and 16:8 L:D.

### Effect of food presence on spatial distribution of predators

In this study we evaluated the impact of the presence of food on the spatial distribution of *N*. *tenuis* and *M*. *pygmaeus* developing on tomato (T) and sesame (S) plants with or without *E*. *kuehniella* eggs (E). Four combinations were made in different cages (30 x 30 x 60 cm covered with fine nylon mesh) including (1) Eggs on both plants (T&S-E); (2) Eggs only on the tomato plant (T-E); (3) Eggs only on the sesame plant (S-E) and (4) Both plants without eggs (T&S).

For all treatments, both predators and plants were simultaneously present in the cages and only the position of food (*E*. *kuehniella* eggs) varied (on the tomato and/or on the sesame plant). An average of 100 eggs of *E*. *kuehniella* were deposited on the leaves with a fine brush. At each observation, the distribution of predators on the different plants (lower, medium and upper parts of the plant) in the cage was noted. The upper, medium and lower parts were represented respectively by the first, second and third foliar stage of the plants and the proportion of each predator was monitored per day over 48 hours. A first observation was made in the morning (7:00 to 11:00 a.m.) and the second in the afternoon (4:00 to 7:00 p.m.), reported as the periods of high activity of *M*. *pygmaeus* [60]. For each treatment (T&S-E; T-E; S-E and T&S), 6 replicates were performed with 10 adult individuals per replicate (5 adults of *N*. *tenuis* and 5 adults of *M*. *pygmaeus*). These experiments were conducted under the same laboratory conditions described previously.

### Statistical analyses

All statistical analyses were carried out using R software (R Development Core Team, version 3.3.3). A separate Generalized Linear Model (GLM) with a binomial error distribution was used to test for the effects of the presence of *M*. *pygmaeus* (absence or presence), the presence of *N*. *tenuis* (absence or presence) and the presence of sesame as a companion plant (absence or presence) on the proportion of hatched *T*. *absoluta* eggs. The effects of the presence of *M*. *pygmaeus* (absence or presence and the presence of sesame as a companion plant (absence or presence)) on the number of necrotic rings caused by *N*. *tenuis* was analyzed using GLM with a Poisson error distribution. A series of linear models (LMs) were used to test for the effects of predatory species, prey availability on the crop plant (tomato plant alone or supplemented with alternative prey), prey availability on the companion plant (sesame plant alone or supplemented with alternative prey), and/or the plant part (lower, medium, upper) on (i) the proportion of predators found on the tomato plants (vs. the sesame plant), and (ii) the number of predators found on the different parts of the tomato and sesame plants. The use of LMs for these tests was appropriate since the dependent variables and the model residuals followed a normal distribution when using a Shapiro–Wilk test and a visual interpretation of quantile–quantile plots. Since at least 2 factors had a significant effect on each response variable, and multi-comparison tests were performed considering each treatment independently (’multcomp’ package, Tukey method).

## Results

### Influence of companion plant and predators on *Tuta absoluta* predation and tomato protection

**Fig 1:**
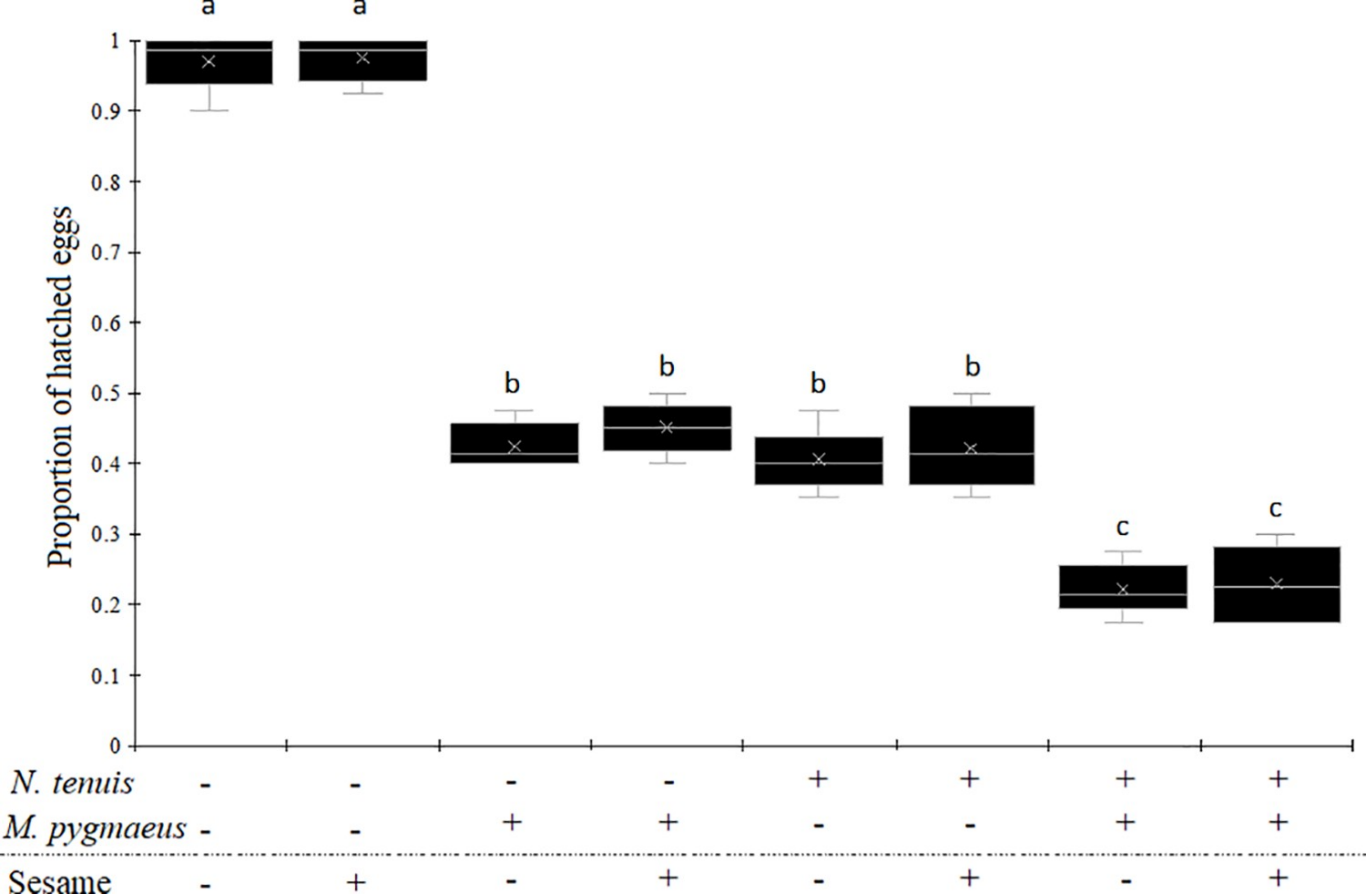
Proportion of hatched eggs of *Tuta absoluta* according to the presence or absence of *Nesidiocoris tenuis* and/or *Macrolophus pygmaeus* and the presence or absence of sesame plant. The ‘+’ indicates the presence and the ‘-’ indicates the absence. Boxplot followed by the same lower case letter did not differ significantly.

The proportion of hatched eggs of *T*. *absoluta* varied significantly depending on the simultaneous presence or not of *N*. *tenuis* and *M*. *pygmaeus* (Table 1, Fig 1). It was twice lower in the presence of *N*. *tenuis* or *M*. *pygmaeus* compared to the condition without predators (Fig 1). When both predators were present simultaneously, this proportion was four times lower compared to the treatment without predators. The presence of sesame did not modulate the egg hatching (Table 1).

**Table 1:** Results of the linear (F value) and Generalized Linear Model (χ^2^ deviance).

Variable to explain	Explanatory variables	F value or χ^2^ deviance	P value
	Presence of *N*. *tenuis*	294.6	< 0.001
**Proportion of hatched eggs**	Presence of *M*. *pygmaeus*	310.0	< 0.001
	Presence of sesame	0.5	0.484
**Number of necrotic rings**	Presence of *M*. *pygmaeus*	5.0	0.025
	Presence of sesame	15.4	< 0.001
**Proportion of predators**	Predator species	74.4	< 0.001
**on tomato plant**	Prey availability on crop plant	37.4	< 0.001
	Prey availability on companion plant	29.0	< 0.001
	Predator species	19.7 / 20.5	< 0.001 / < 0.001
**Number of predators**	Prey availability on crop plant	8.8 / 11.2	0.004 / 0.001
**(on tomato / on sesame)**	Prey availability on companion plant	8.8 / 8.7	0.004 / 0.004
	Plant part	18.1 / 41.9	< 0.001 / < 0.001

### *Nesidiocoris tenuis* phytophagy

**Fig 2:**
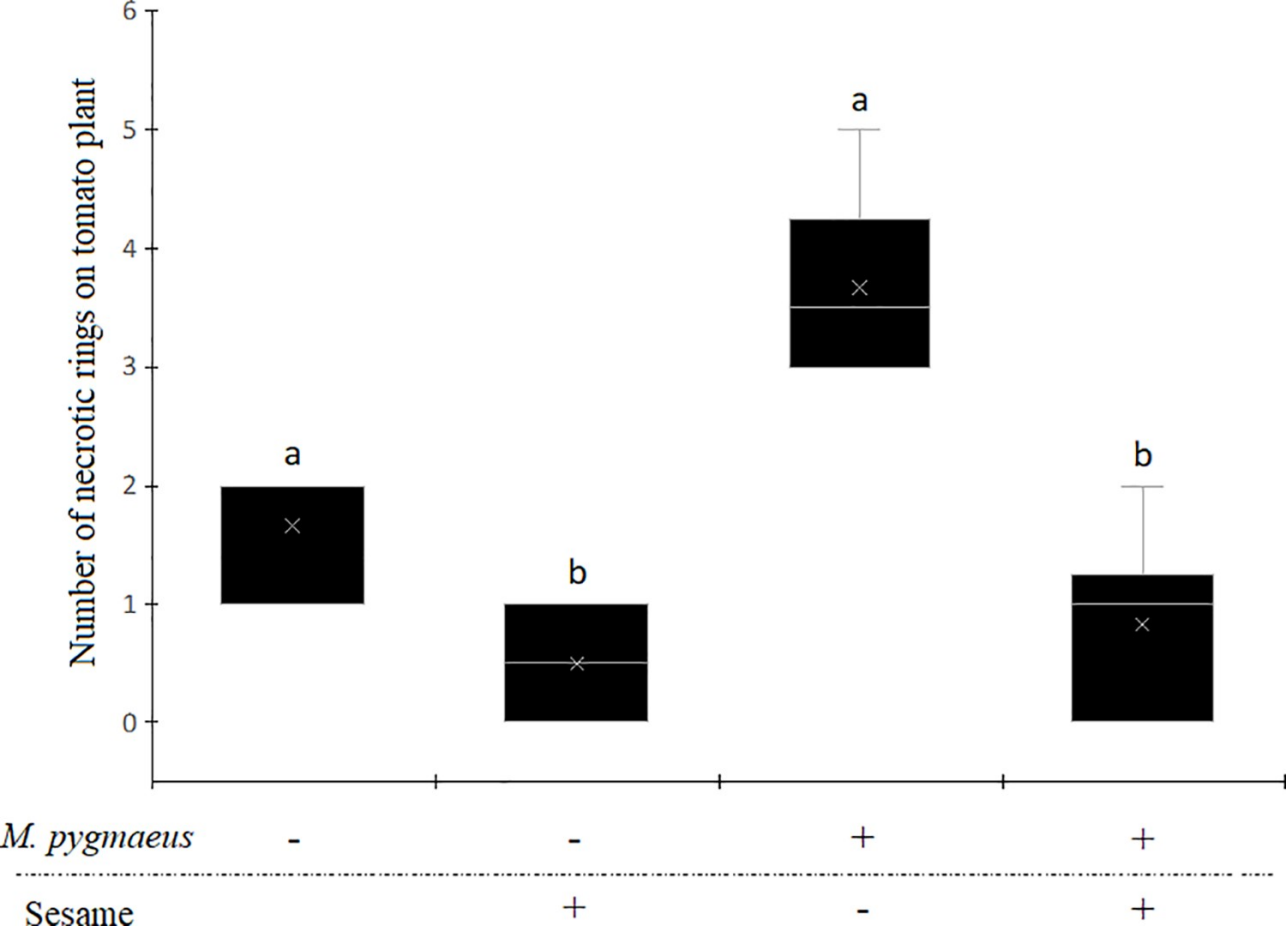
Number of necrotic rings caused by *Nesidiocoris tenuis* on the tomato plant according to the presence or absence of *Macrolophus pygmaeus* and the presence or absence of sesame plant. The ‘+’ indicates the presence and the ‘-’ indicates the absence. Boxplot followed by the same lower case letter did not differ significantly.

The number of necrotic rings caused by *N*. *tenuis* on tomato plants significantly depended on the presence of either *M*. *pygmaeus* or sesame (Table 1, Fig 2). Without *M*. *pygmaeus*, the number of necrotic rings was 3.3 times lower in presence of sesame than in absence of the companion plant. When both predators were present simultaneously, the number of necrotic rings was 4.4 lower in presence of sesame than in absence of the companion plant. The number of necrotic rings was 2 times higher in presence of both predators compared to the treatment when *M*. *pygmaeus* was absent.

### Effect of food presence on spatial distribution of predators

**Fig 3:**
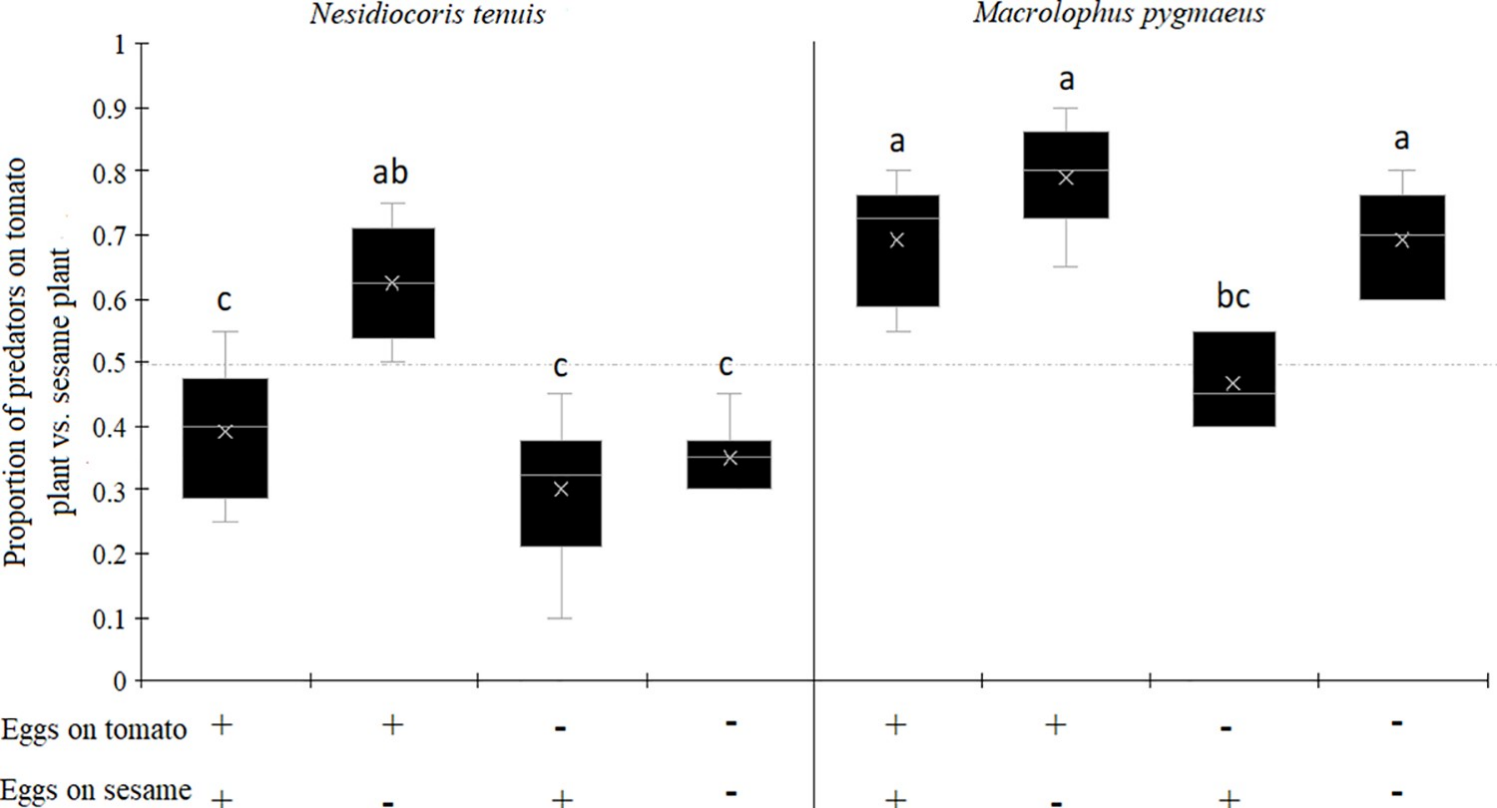
Proportion of predators (*Nesidiocoris tenuis* or *Macrolophus pygmaeus*) on tomato vs sesame plants according to the presence or absence of *Ephestia kuehniella* eggs on the tomato or the sesame plant. The ‘+’ indicates the presence and the ‘-’ indicates the absence. Boxplot followed by the same lower case letter did not differ significantly.

The proportion of predators on the tomato plant vs. the sesame plant varied significantly according to the predator species, the prey availability on the host crop plant and the prey availability on the companion plant (Table 1, Fig 3). *Nesidiocoris tenuis* preferred tomato when the prey was only on tomato (proportion of *N*. *tenuis* on tomato = 69%). In contrast, the proportion of *N*. *tenuis* was lower when the prey was on both plants (39%), only on sesame (30%) or without prey on both plants (35%). *Macrolophus pygmaeus* preferred tomato than sesame plant when prey was present on both plants, only on tomato or in absence of prey on both plants with the proportions of 69, 80 and 69%, respectively. This proportion was low (less than 50%) when the prey was only on sesame plant.

**Fig 4:**
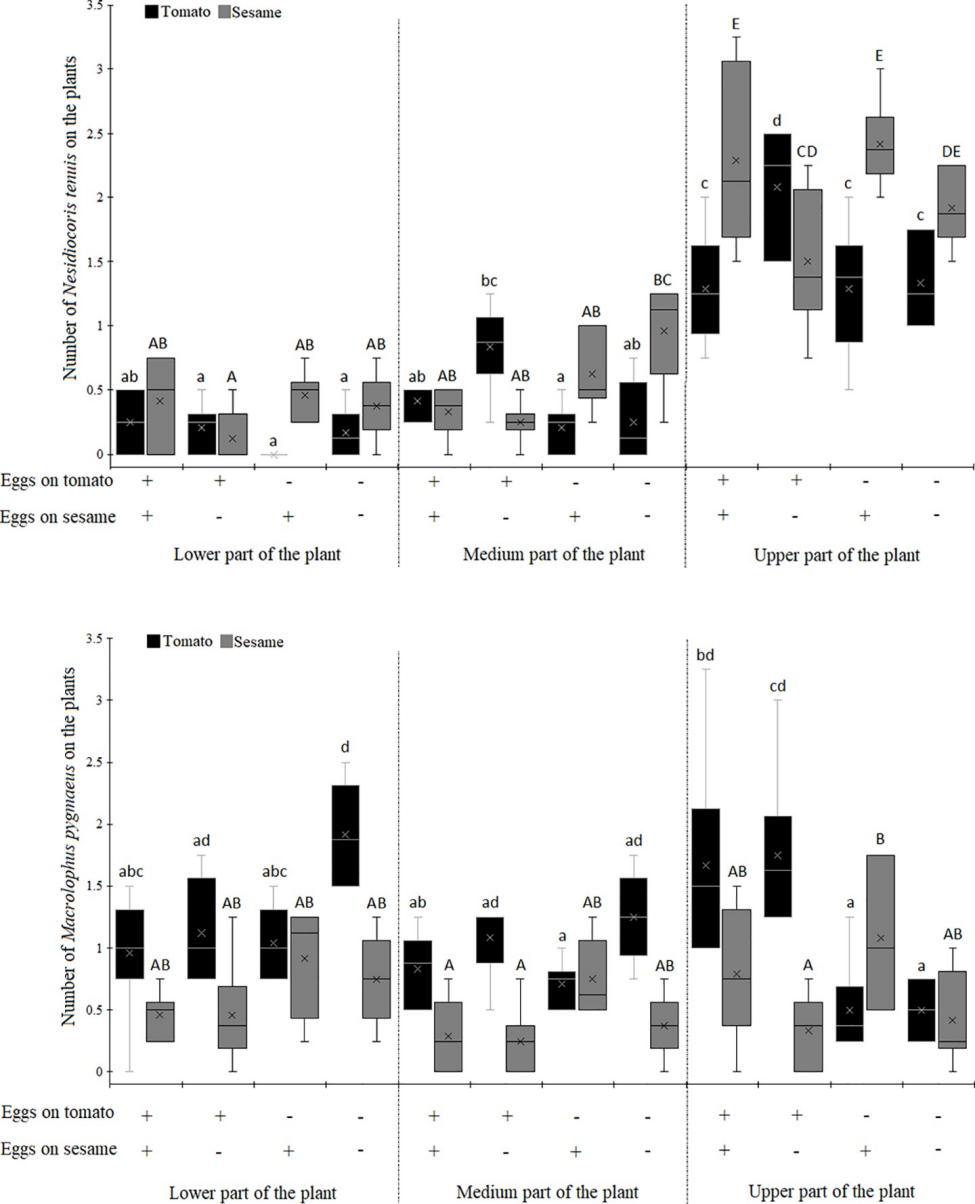
A. Number of *Nesidiocoris tenuis* on the different parts of the tomato or sesame plants depending on the presence or absence of *Ephestia kuehniella* eggs on the tomato or the sesame plant. The ‘+’ indicates the presence and the ‘-’ indicates the absence. Boxplot followed by the same lower or upper case letter did not differ significantly with respect to plant species (tomato and sesame, respectively). B. Number of predators *Macrolophus pygmaeus* on the different parts of the tomato or sesame plants depending on the presence or absence of *Ephestia kuehniella* eggs on the tomato or the sesame plant. The ‘+’ indicates the presence and the ‘-’ indicates the absence. Boxplot followed by the same lower or upper case letter did not differ significantly with respect to plant species (tomato and sesame, respectively).

The number of predators established on the tomato and sesame plants varied significantly according to the predator species, the prey availability on the host crop plant, the prey availability on the companion plant and the part of plant (Table 1, Fig 4A and 4B). *Nesidiocoris tenuis* significantly preferred the upper part of the tomato and sesame plants compared to the medium and lower parts, whereas no difference was observed between the medium and the lower parts (Fig 4A). The presence of *N*. *tenuis* on the lower part of the tomato or sesame plant did not vary according to prey availability on the tomato or sesame plant. The number of *N*. *tenuis* individuals observed on the medium part of the tomato plant was higher when the alternative prey was provided only on the tomato plant than when the alternative prey was present only on the sesame plant. By contrast, the number of *N*. *tenuis* individuals on the medium part of the sesame plant did not vary according to availability of the alternative prey. Finally, the presence of *N*. *tenuis* on the upper part of the tomato varied according to prey availability as a higher number of individuals were observed on tomato plants when the alternative prey was provided only on the tomato plant compared to when alternative food was provided on the sesame plant, on both plants or was not provided. When considering the upper part of the sesame plant, the number of individuals observed was higher when the alternative prey was provided only on the sesame plant or on both plant species than when alternative food was provided only on the tomato plant.

The number of *M*. *pygmaeus* did not vary among the different tomato or sesame plant parts (Fig 4B). The number of individuals in the lower part of the tomato plant was higher when no alternative prey was provided compared to when alternative prey was provided on the sesame plant only and on both plant species. By contrast, the number of *M*. *pygmaeus* individuals did not vary when they were developed on the lower part of sesame. The number of individuals was similar in the different treatments (when alternative prey was provided on the tomato plant, on the sesame plant, on both plants or was not provided) for predators in medium part of the plants. Finally, the number of individuals in the upper part of the tomato plant was higher when the alternative prey was provided on the tomato plant only or on both plant species than when the alternative prey was provided on the sesame plant only or was not provided at all. By contrast, the number of individuals in the upper part of the sesame plant was higher when the alternative prey was provided on the sesame plant than when the alternative prey was provided on the tomato plant only.

## Discussion

Competition for the same prey in coexisting predators is expected to increase phytophagy of omnivorous predators, induce intraguild predation, and increase their effectiveness as biological control agents. However, the combined action of coexisting predators was also reported to be beneficial in controlling pests. In such a context, we evaluated the impact of the presence of a companion plant on predator localization, effectiveness in controlling pests and reducing damage to cultivated plants. Intraguild predation occurs when two species that share a common prey resource also engage in trophic interaction with each other (e.g., predation) [61–63]. The results of our study did not provide evidence of intraguild predation between the predators *N*. *tenuis* et *M*. *pygmaeus*. This could be due to several factors such as the stage (adult) of the individuals in competition, the presence of other food sources (plants), the duration of the competition (2 days) or the complexity of the habitat (sesame + tomato). Indeed, the relative size and higher mobility of individuals is an important determinant of predator-predator interaction [64, 65]. For example, adult females of *N*. *tenuis* are known to inflict a high mortality rate only on young nymphs but not on adults of *M*. *pygmaeus* in the absence of alternative prey [66]. In addition, the ability of predators to consume food resources (plants) other than the shared resource (prey) can reduce competitive interaction and promote coexistence [32, 65, 67].

In our study we observed a much lower hatching rate of *T*. *absoluta* eggs when both predators were present together than when only one predator species was released. This could be due to competition for the same prey (*T*. *absoluta* eggs), the voracity of predators or the distribution of predators on all plant parts. Such behaviors could initially reduce egg hatching but also improve crop protection against this pest. Walzer et al. [68] indicated that bean protection against *Tetranycus urticae* Koch (Acari: Tetranychidae) was improved when the predators *Phytoseiulus persimilis* Athias-Henriot (Acari: Phytoseiidae) and *Neoseiulus californicus* McGregor (Acari: Phytoseiidae) coexisted. Also, the complementary occupation by both predators of different parts of the plant i.e., *N*. *tenuis* in the upper part and *M*. *pygmaeus* on all parts could increase their efficacy on the whole plant. Moreover, according to Lucas and Alomar [69], the level of whitefly predation was higher in the lower part of the plant when *D*. *tamaninii* and *M*. *pygmaeus* coexisted than when there was only one species. Moreno-Ripoll et al. [70] recommended the combined use of *Eretmocerus mundus* Mercet (Hymenoptera: Aphelinidae), *M*. *pygmaeus* and *N*. *tenuis* in order to improve *Bemisia tabaci* Gennadius (Hemiptera: Aleyrodidae) biological control. In a review study on generalist predators, Symondson et al. [29] showed a significant reduction of pests (Mollusca, Diptera, Lepidoptera, Coleoptera, Acari, Hemiptera and others) under experimental conditions by these predators in 79% of 52 studies consulted and yield increased significantly for 65% of the 26 cases where effects on plants were measured. Our results also provide evidence that the presence of a companion plant such as *S*. *indicum* does not influence mirid predation on *T*. *absoluta* eggs (consistent with Biondi et al. [42]). Sesame could therefore have a positive impact on the biological control of *T*. *absoluta* by *N*. *tenuis* and *M pygmaeus*. The positive influence of this plant in improving the biocontrol performance of predatory mirid *Cyrtorhinus lividipennis* Reuter (Hemiptera: Miridae) was already demonstrated on rice pests including *Nilaparvata lugens* Stål (Hemiptera: Delphacidae), *Marasmia Patnalis* Bradley (Lepidoptera: Pyralidae) and *Cnaphalocrocis medinalis* Guenée (Lepidoptera: Crambidae) [71].

Damage induced by *N*. *tenuis* on tomato plants was also mitigated by sesame plant presence: a higher number of necrotic rings was observed when predators were combined on the tomato plant in the absence of a sesame plant. This could be due to competition for the same prey causing a rapid drop in the availability of this prey and encouraging the repeated feeding of mirids on the only food source present, i.e., the tomato plant. When the level of prey in the crop is low, the phytophagy activity of *N*. *tenuis* may be of critical importance [36, 37]. According to Sanchez [72], the damage caused by *N*. *tenuis* to the tomato plant is inversely related to the abundance of prey. Damage can also be explained by the predators’ need for plant-based nutrients in order to optimize digestion and/or assimilation of its prey [73]. The intensity of damage varies according to the availability of prey and the presence of a plant resource other than a cultivated plant [65, 67]. This could explain our observation that the number of necrotic rings was reduced when the sesame plant was added. This also implies that the presence of sesame reduces the damage caused by *N*. *tenuis* to tomato plants. Indeed, *N*. *tenuis* prefers sesame to tomato plants for its nutrition and development [41, 42, 74]. In other words, this predator prefers to feed on sesame rather than on tomato plants when offered the choice, as was the case in our study conditions. Sesame could be a better source of plant nutrients than tomato. A study by Naselli et al. [75] showed that sesame plants emitted lower amounts of hydrocarbon monoterpenes but higher levels of oxygenated terpenes than tomato plants. While hydrocarbon terpenes are known to have insect pest repellent properties [76], oxygenated monoterpenes and Green Leaf Volatiles (GLV) compounds have been shown to play a role in attracting predatory mirids [75, 77]. Also, in addition to having no influence on egg predation, sesame considerably reduces *N*. *tenuis* damage to tomato plants. According to Gillespie and McGregor [73] and Biondi et al. [42], this companion plant is a good food source that is effective in disrupting the phytophagy activity of this mirid on the cultivated plant without influencing predation. In this study, *N*. *tenuis* preferred the sesame to the tomato plant (except when food was provided only on the tomato plant) and preferred the upper part of the plants, whereas *M*. *pygmaeus* preferred the tomato to the sesame plant (except when food was provided only on the sesame plant) and did not prefer a plant part. The presence of sesame did not reduce the efficacy of *N*. *tenuis* or *M*. *pygmaeus* in consuming *T*. *absoluta* eggs (the proportion of hatched eggs decreased when *N*. *tenuis*, *M*. *pygmaeus* or both predators were present) and clearly reduced the number of necrotic rings when *N*. *tenuis* or both predators were present simultaneously.

In addition, the high proportion of *N*. *tenuis* on the sesame plant and of *M*. *pygmaeus* on the tomato plant indicates that both predators have opposite preferences for plant species. This is not a surprise because *N*. *tenuis* is considered an important pest in sesame cultivation in India and Japan [41]. *Nesidiocoris tenuis* was more attracted to this plant than *Dittrichia viscosa* for its reproduction [42]. According to Nakahishi et al. [41], sesame can be an insectary plant for this predator. *Macrolophus pygmaeus* was more present on tomato than sesame. Tomato plants could be a better food supplement than sesame for this mirid. In crops, this bug is mainly observed on solanaceous plants, more particularly on tomatoes and eggplant. *Macrolophus pygmaeus* develops well on tomato plants even when preys are absent [78]. The high proportion of *N*. *tenuis* on the upper part of the plant and of *M*. *pygmaeus* on the entire plant also indicates different preferences in terms of occupation of different parts of plants. *Nesidiocoris tenuis* is usually present in the upper part of plants while *M*. *pygmaeus* explores the lower leaves [32, 79]. The difference between our results and those of Perdikis et al. [32] on the distribution of *M*. *pygmaeus* could be due to the relative size of the individuals and the number of individuals combined (16 nymphs) in their experience. Predators were distributed in our experimental conditions such that both species encountered each other more often and could not avoid each other completely. Moreover, in a similar experiment, Perdikis et al. [32] observed that one *N*. *tenuis* and one *M*. *pygmaeus* could meet 5 times in 30 minutes for more than 3 seconds. Although avoidance mechanisms are often not clearly identified [80], the avoidance of hetero specific competitors is not always systematic [81]. Our results hint that by occupying different parts of the plant neither predator was in competition. Generally, avoidance behavior occurs between closely related species with a marked overlap in diet i.e., between species of specialized predators [82]. Moreno-Ripoll et al. [66] showed that the distribution of these predators was not modified when they were associated or not. The occupation of different strata on plants by each species allows for better coverage of the plant by predators. This could therefore have important implications for biological control as the potential activity of the predators thus distributed may be complementary [32, 69, 83, 84]. The results of the present study could enable optimizing biological control methods against *T*. *absoluta*, although multiple other factors should be considered to achieve fully effective and sustainable Integrated Pest Management (IPM) strategies [18, 85, 86].

## Supporting information

S1 Data (XLSX)
